# Coagulation Management of Critically Bleeding Patients With Viscoelastic Testing Presented as a 3D-Animated Blood Clot (The Visual Clot): Randomized Controlled High-Fidelity Simulation Study

**DOI:** 10.2196/43895

**Published:** 2023-10-12

**Authors:** Clara Castellucci, Amos Malorgio, Alexandra Dinah Budowski, Samira Akbas, Michaela Kolbe, Bastian Grande, Julia Braun, Christoph B Noethiger, Donat R Spahn, David Werner Tscholl, Tadzio Raoul Roche

**Affiliations:** 1 Institute of Anaesthesiology, University Hospital Zurich University of Zurich Zurich Switzerland; 2 Simulation Centre, University Hospital Zurich University of Zurich Zurich Switzerland; 3 Department of Epidemiology Epidemiology, Biostatistics and Prevention Institute University of Zurich Zurich Switzerland; 4 Department of Biostatistics Epidemiology, Biostatistics and Prevention Institute University of Zurich Zurich Switzerland

**Keywords:** avatar technology, coagulation management, high-fidelity simulation, point-of-care testing, thrombelastography, user-centered design, Visual Clot

## Abstract

**Background:**

Guidelines recommend using viscoelastic coagulation tests to guide coagulation management, but interpreting the results remains challenging. Visual Clot, a 3D animated blood clot, facilitates interpretation through a user-centered and situation awareness–oriented design.

**Objective:**

This study aims to compare the effects of Visual Clot versus conventional viscoelastic test results (rotational thrombelastometry [ROTEM] temograms) on the coagulation management performance of anesthesia teams in critical bleeding situations.

**Methods:**

We conducted a prospective, randomized, high-fidelity simulation study in which anesthesia teams (consisting of a senior anesthesiologist, a resident anesthesiologist, and an anesthesia nurse) managed perioperative bleeding scenarios. Teams had either Visual Clot or ROTEM temograms available to perform targeted coagulation management. We analyzed the 15-minute simulations with post hoc video analysis. The primary outcome was correct targeted coagulation therapy. Secondary outcomes were time to targeted coagulation therapy, confidence, and workload. In addition, we have conducted a qualitative survey on user acceptance of Visual Clot. We used Poisson regression, Cox regression, and mixed logistic regression models, adjusted for various potential confounders, to analyze the data.

**Results:**

We analyzed 59 simulations. Teams using Visual Clot were more likely to deliver the overall targeted coagulation therapy correctly (rate ratio 1.56, 95% CI 1.00-2.47; *P*=.05) and administer the first targeted coagulation product faster (hazard ratio 2.58, 95% CI 1.37-4.85; *P*=.003). In addition, participants showed higher decision confidence with Visual Clot (odds ratio 3.60, 95% CI 1.49-8.71; *P*=.005). We found no difference in workload (coefficient –0.03, 95% CI –3.08 to 2.88; *P*=.99).

**Conclusions:**

Using Visual Clot led to a more accurate and faster-targeted coagulation therapy than using ROTEM temograms. We suggest that relevant viscoelastic test manufacturers consider augmenting their complex result presentation with intuitive, easy-to-understand visualization to ease users’ burden from unnecessary cognitive load and enhance patient care.

## Introduction

Several guidelines recommend applying viscoelastic coagulation tests to guide transfusions and the administration of coagulation factors in cases of severe bleeding [1,2]. Compared to standard laboratory coagulation tests, thromboelastography (TEG) and rotational thrombelastometry (ROTEM) have proven to be more time- [3] and cost-efficient [4]. Furthermore, viscoelastic-guided transfusion algorithms reduce inadequate blood transfusions and lower overall mortality [5]. However, despite the apparent importance, general acceptance, and expanding usage, the correct and timely interpretation of viscoelastic coagulation tests remains challenging [6]. Prolonged or incorrect analysis interrupts and impairs workflow, which may lead to diagnostic errors and subsequently to incorrect or inappropriate treatment.

Consequently, we developed the Visual Clot technology to simplify the visualization of viscoelastic test results. This animated, 3D blood clot illustrates raw TEG and ROTEM data while considering user-centered and situation-awareness design aspects. Visual Clot displays various clot components as present or absent based on empirical TEG and ROTEM cutoff values without taking the final decision away from the user. In an earlier prospective, computer-based study, Visual Clot supported anesthesia and intensive care physicians in Germany and Switzerland by improving their therapeutic choices in simulated coagulation management scenarios. In addition, physicians made decisions faster, had more confidence in the selected therapy, and experienced less workload while managing hypothetical bleeding scenarios [7]. After their initial experiences, the same physicians considered Visual Clot intuitive, easy to learn, and useful for decision-making [8]. In a second computer-based study, Visual Clot enabled medical students without previous experience interpreting viscoelastic tests to administer the correct products for appropriate coagulation management [9]. Further, an eye-tracking study showed that physicians who were unfamiliar with Visual Clot spent less time viewing the results while deciding 4 times faster on the correct coagulation therapy than with a conventional viscoelastic test display [10].

Here we apply this new technology for the first time in a high-fidelity simulation environment, a valuable way to test a noncertified product very close to clinical reality [11,12]. Using simulation as investigative methodology, we evaluated the performance of anesthesia teams in managing simulated critically bleeding scenarios using Visual Clot or the conventional presentation of viscoelastic test results as ROTEM temograms. We hypothesized that anesthesia teams using Visual Clot would treat patients faster and more often correctly. In addition, we investigated perceived decision confidence and workload.

## Methods

### Overview

We present an investigator-initiated, prospective, randomized, high-fidelity simulation study conducted over 3 consecutive weeks in January and February 2022 at the Simulation Center of the University Hospital Zurich as part of the annual simulation training program of the Institute of Anesthesiology, University Hospital Zurich, Switzerland.

**Figure 1:**
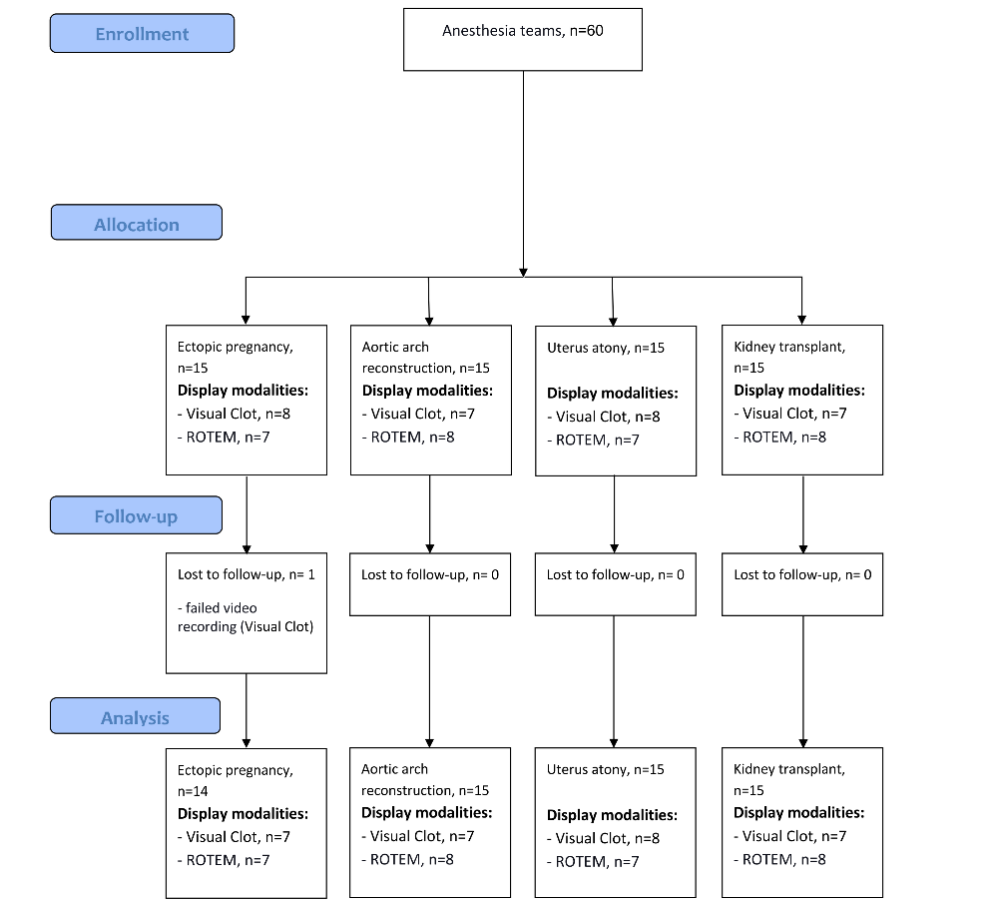
The study flow diagram shows enrolled anesthesia teams, the assignment to the different scenarios, and the display modalities used, as well as excluded teams and analyzed data. ROTEM: rotational thrombelastometry.

We randomly allocated teams (consisting of a senior anesthesiologist, a resident anesthesiologist, and an anesthesia nurse) to the intervention group, “Visual Clot,” or the control group, “standard ROTEM temograms” (Figure 1). Subsequently, we evaluated the coagulation management of the anesthesia teams during standardized simulated critical bleeding scenarios. Participants were scheduled for simulation training 1 month in advance by our staff manager and were relieved of their clinical duties during this time.

The study institution follows a center-wide coagulation management algorithm (Multimedia Appendix 1) in which general measures (eg, warming) and targeted measures (eg,* *clotting factor concentrates)*,* using ROTEM and clotting factor analysis, are indicated to maintain homeostasis during ongoing bleeding.

### Visual Clot and ROTEM Temograms

**Figure 2:**
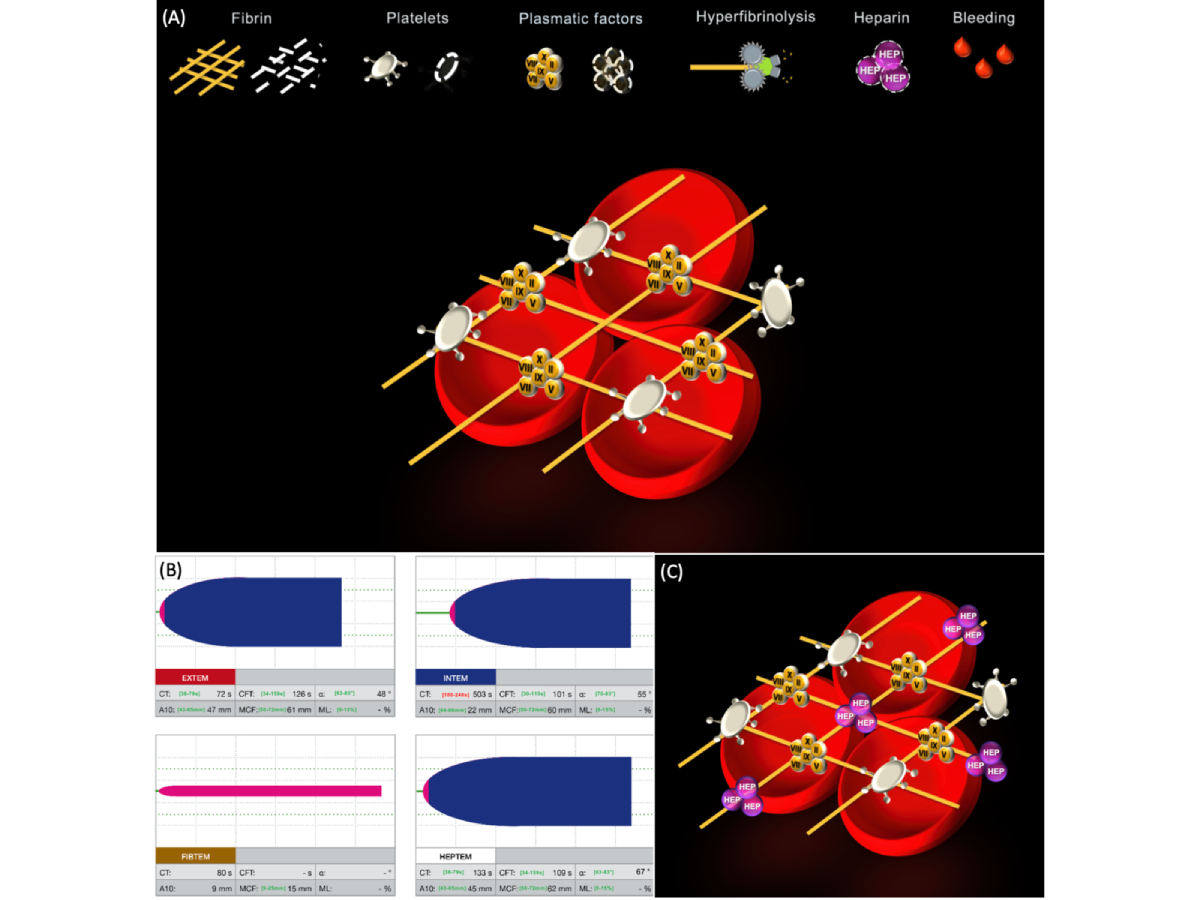
Visual Clot. (A) A normal Visual Clot with no coagulation pathologies. The legend describes the individual icons. If fibrin, platelets, or plasmatic factors are missing, they are shown as dashed lines. In the case of hyperfibrinolysis, the harvester disrupts the fibrin scaffold, and a heparin effect is indicated by the corresponding icon. The bleeding effect occurs if anything is abnormal. (B) ROTEM temograms of EXTEM (tests the extrinsic pathway), INTEM (tests the intrinsic pathway), FIBTEM (tests the fibrinogen function), and HEPTEM (tests the heparin effect) channels. Due to a residual effect of heparin, the clotting time is prolonged in INTEM but normal in HEPTEM. (C) Visual Clot showing a residual effect of heparin.

The anesthesia team received the Visual Clot animation or standard ROTEM temograms to guide coagulation management (Figure 2). In brief, Visual Clot displays fibrin, platelets, and plasmatic factors in a schematic animation, correlating with data from the viscoelastic tests. In case of deficiency, the hemostatic components appear in flashing, dotted lines, while newly appearing symbols indicate a surplus of heparin or hyperfibrinolysis. The Visual Clot instructional video (Multimedia Appendix 2) explains the function and display of Visual Clot in detail.

### Simulated Scenarios

The critical bleeding scenarios included an ectopic pregnancy with a progressive increase of free pelvic fluid, an aortic arch reconstruction with insufficient heparin reversal, uterine atony after the cesarean delivery of twins, and a kidney transplant leading to a massive hemorrhage due to surgical complications. Each case lasted precisely 15 minutes. After 5 minutes from the beginning of each case, the teams received either Visual Clot or corresponding ROTEM temograms, presenting the current coagulation status of the patient. Following the coagulation management algorithm, teams had to administer several predefined therapies to stop the bleeding and correctly solve the scenario. Detailed case descriptions for every scenario and the shown Visual Clots and ROTEM temograms can be found in Multimedia Appendix 3.

Each team participated in only 1 critical bleeding scenario with either Visual Clot or ROTEM temograms at their disposal. We randomized which scenario was processed by which team and which modality (Visual Clot and ROTEM Temograms) was used [13].

### Simulation Environment and Equipment

We conducted this study in our hospital’s simulation center, which was set up as a standard operating room [12,14]. The setup included a state-of-the-art patient simulator, SimMan 3G (Laerdal Medical), with airway management equipment and patient monitoring. To further increase the simulation’s fidelity, we used real drugs, and members of the simulation center acted as surgeons in the scenarios. Study investigators and the simulator technician were located in an adjacent room, separated by a 1-way mirror. An overview of the simulation rooms can be found in Multimedia Appendix 4.

### Simulation Procedure

On each simulation day, the educators welcomed participants and spent approximately one hour establishing an inviting and engaging learning atmosphere, providing orientation to the learning objectives and training details, as well as familiarizing them with the simulation equipment. These thorough briefings improve simulation performance and reduce participant aversion or defensiveness, which otherwise can be seen in the context of health care simulations [12,15]. Furthermore, participants received a brief introduction explaining the study and the interpretation of Visual Clot through a short video (Multimedia Appendix 2), which took approximately 5 minutes.

After completing the orientation, teams filled out a short demographic survey on an iPad (Apple Inc) using the iSurvey app (Harvest Your Data) [16]. We then briefed the team on the upcoming scenario, highlighting the patient’s medical history, ongoing or planned surgery, and any special features related to medications or previous lab results (Multimedia Appendix 3).

We programmed each of the 4 critical bleeding scenarios by predefining the timing of vital sign changes and events to achieve standardization of events and scripts throughout the study. After 5 minutes into the scenario, the team received either Visual Clot or ROTEM temogram (Multimedia Appendix 3). All scenarios included the role of a surgeon, who adhered to a script and received instructions through an earpiece from the investigators to demonstrate consistent and realistic behavior in a standardized way. Upon completion of the simulated case, participating team members were asked to fill out a questionnaire that captured their perceived decision confidence, workload, and scenario fidelity. After a debriefing, the following team proceeded with the next simulation.

### Outcomes

In this study, we distinguished between therapies that can be derived directly from the assessment of the viscoelastic test (targeted coagulation therapy) and other general coagulation management measures.

Targeted coagulation therapy is defined as the administration of coagulation products whose indication can be derived solely from the Visual Clot or ROTEM temograms in the simulated scenarios. Overall coagulation therapy includes all therapeutic measures indicated for coagulation management in the respective scenario, including, but not limited to, those measures that can be derived from the Visual Clot or ROTEM temograms. Table 1 lists all 4 scenarios, including which measures are considered targeted coagulation therapy and which measures are summarized under overall coagulation therapy.

The primary outcome was correct targeted coagulation therapy measured as the number of correct and complete therapeutic actions resulting solely from Visual Clot or ROTEM temograms interpretation.

Secondary outcomes were defined as time to targeted coagulation therapy and correct overall coagulation therapy. The former was measured as the time in seconds from receiving Visual Clot or ROTEM temograms (standardized 5 minutes after scenario start) to administering the first correct targeted coagulation product. The latter we measured as the number of all correct therapeutic actions regarding coagulation management for the corresponding scenario. We abstained from assessing overall coagulation therapy, as teams had limited time and the number of necessary tasks to perform differed in each scenario.

Finally, we examined participants’ perceived decision confidence (binary as confident or unconfident) and perceived workload during the simulations [17,18]. We measured the workload using the raw National Aeronautics and Space Administration Task Load Index (NASA TLX). This questionnaire is validated to assess perceived workload by answering 6 specific questions ranging from 0 (very low) to 100 (very high workload). The total score is determined from the arithmetic mean of all partial results without weighing them [18-20].

Additionally, we asked the participants who used Visual Clot to rate 4 general statements about this technique on a 4-point Likert scale (Multimedia Appendix 5).

**Table 1:** Targeted and overall coagulation therapy per scenario. Please note that the items under targeted coagulation therapy are also part of the overall coagulation therapy. Please note that tranexamic acid was added to the overall coagulation therapy section because it is administered empirically in the coagulation algorithm of the study institution, even before rotational thrombelastometry (ROTEM) results are available. Of course, hyperfibrinolysis, and thus the indication for tranexamic acid administration, can also be detected in ROTEM.

			Ectopic pregnancy		Aortic arch reconstruction		Uterine atony		Kidney transplant
**Targeted coagulation therapy**									
	Fibrinogen concentrate	✓		✓		✓		✓	
	Platelets			✓		✓		✓	
	Protamine			✓					
	4-factor prothrombin complex							✓	
**Overall coagulation therapy**									
	Warming	✓		✓		✓		✓	
	Calcium	✓		✓		✓		✓	
	Tranexamic acid	✓				✓		✓	
	Packed red blood cells							✓	
	Factor XIII concentrate							✓	
	Fluids	✓						✓	
	Fresh frozen plasma							✓	

### Video-Based Analysis

To analyze the simulations, we used composite, synchronized videos showing 3 different angles of the simulation as well as the vital signs on the monitor (Multimedia Appendix 6). Using these videos and an Excel (Microsoft Corp) spreadsheet, we entered the number of seconds between the start of the simulation and the time previously specified tasks were executed. These included the time points for executing targeted and overall coagulation therapy measures. We only included the times of correctly performed measures.

### Statistical Analysis

Note that the time outcomes (time to targeted coagulation therapy and time to verbalizing targeted coagulation therapy) and the performance outcomes (correct targeted coagulation therapy and correct overall coagulation therapy) are measured on a team level, taking any bias of team members participating in more than 1 scenario into account. The outcomes, perceived decision confidence, perceived workload, and the statement results are measured at the individual participant level. As multiple scenarios, which should represent a wide display of critical bleeding cases, can influence the comparability of results, we included the respective scenario in the regression models so that the influence of the Visual Clot or ROTEM can be interpreted independently from the case.

For descriptive statistics, we show medians and ranges for continuous data and numbers and percentages for categorical data.

For the time outcomes (time to targeted coagulation therapy and time to verbalizing targeted coagulation therapy), we use Cox regression models, adjusted for the 4 scenarios, to see if there is a difference between the modalities (Visual Clot or ROTEM temograms).

To analyze the outcomes, correct targeted coagulation therapy and correct overall coagulation therapy, we use Poisson regression models adjusted for the 4 scenarios, with the maximal number of correct therapy actions as an offset. Note that we treat the teams as independent, although a participant might be a member of different team.

For the binary decision confidence variable, we use a mixed logistic regression model with a random intercept per participant. To analyze workload (NASA TLX total score), we use a linear mixed model with a random intercept for each participant. Please note that for these 2 secondary outcomes, we examined data per participant and not an averaged value per team. Therefore, we opted for the calculation of mixed models that take into account that the same person could be present in more than 1 scenario and thus potentially provide more than 1 answer to the confidence and workload questions.

Following publications on medical statistics [21,22], we interpret a *P* value of more than .10 as providing little or no evidence, a *P* value between .05 and .10 as weak evidence, a *P* value between .01 and .001 as strong evidence, and less than .001 as very strong evidence against the null hypothesis. The advantage of this interpretation, which has become more and more common and is nowadays taught to medical students, is that *P* values are not arbitrarily dichotomized anymore (“significant” vs “not significant”), so very similar *P* values of, say, .049 and .051 lead to totally different decisions, as would be the case with the “classical” interpretation of *P* values in the medical literature. Instead, this strategy allows for an actual interpretation of the *P* value in a quantitative sense (quantifying the evidence against the null hypothesis) while at the same time avoiding the difficulties associated with the statistical theory of the *P* value. This way, it can also be recognized that an effect with a *P* value slightly above the border of significance still has an actual meaning and can be discussed without having to stress that a study might be underpowered.

As the study was conducted as part of the regularly held simulation training at the University Hospital Zurich, Switzerland, no sample size calculation was performed, as the number of participants and the resulting teams were predetermined by the number of planned simulation days.

### Ethical Considerations

The Cantonal Ethics Commission of Zurich, Switzerland, issued a declaration of no objection after reviewing the study protocol (Business Management System for Ethics Committees Number Req-2021-01112). Additionally, each participant gave their written informed consent to use their data, including their understanding that participation in the study was voluntary and not compensated. We adhered to the reporting guidelines for health care simulation research, an extension to the CONSORT (Consolidated Standards of Reporting Trials) and STROBE (Strengthening the Reporting of Observational Studies in Epidemiology) statements [23]. This randomized controlled study was not registered prospectively, given that participants were health care professionals with no patients or drugs involved.

## Results

### Overview

Between January and February 2022, a total of 60 anesthesia teams consisting of a senior physician, a resident physician, and a nurse anesthetist performed 60 high-fidelity simulations. We excluded 1 simulation for technical reasons (failed video recording). In case of unavailability, the senior physician was replaced by an experienced resident physician in 8 simulations. Overall, 2 teams consisted of only 2 participants (resident physician and nurse anesthetist), as no third person was available for the simulation. Most participants agreed or fully agreed (157/178, 88%) that the simulated cases realistically reflected everyday clinical practice. Of the 85 participants, 56 individuals participated in more than 1 scenario on the same day without seeing the same scenario or condition twice. Table 2 provides additional study and participant characteristics. Figure 1 provides the study flow diagram.

**Table 2:** Study and participant characteristics.

			Values
**Study characteristics**			
	Performed simulations, n	60	
	Excluded simulations, n (%)	1 (2)	
	Ectopic pregnancy simulation, n (%)	14 (24)	
	Aortic arch reconstruction simulation, n (%)	15 (25)	
	Uterus atony simulation, n (%)	15 (25)	
	Kidney transplant simulation, n (%)	15 (25)	
	Visual Clot, n (%)	29 (49)	
	ROTEM^a^ temograms, n (%)	30 (51)	
**Participant characteristics**			
	Study participants, n	85	
	Anesthesia teams, n	60	
	Gender female, n (%)	51 (60)	
	Senior physician, n (%)	13 (15)	
	Resident physician, n (%)	32 (38)	
	Nurse anesthetist, n (%)	40 (47)	
	Age of participants (years), median (IQR)	34 (25-60)	
	Work experience of participants (years), median (IQR)	5 (0-33)	
	Self-assessed ROTEM experience (0=novice, 100=expert), median (IQR)	37 (0-100)	

^a^ROTEM: rotational thrombelastometry.

### Correct Targeted Coagulation Therapy

**Figure 3:**
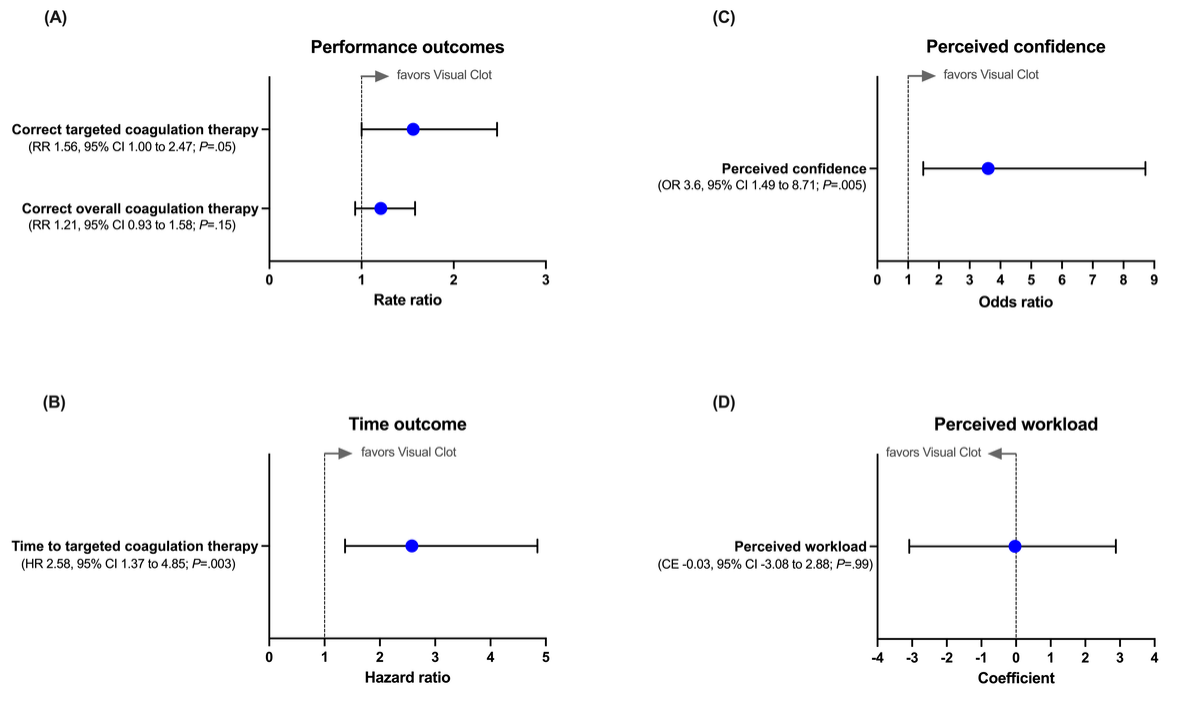
(A) Analysis of time outcomes using an adjusted Cox regression model. The presented bars are hazard ratios (HRs) with 95% CIs. (B) Analysis of performance outcomes using Poisson regression models. The presented bars are rate ratios (RRs) with 95% CIs. (C) Analysis of perceived confidence using a mixed logistic regression model. The presented bar is an odds ratio (OR) with 95% CIs. (D) Analysis of perceived workload using a mixed linear regression model. The presented bar is a coefficient with 95% CIs. CE: coefficient.

For the primary outcome, the Poisson regression model was favorable for Visual Clot with a rate ratio of 1.56 (95% CI 1.00-2.47; *P*=.05) compared with ROTEM temograms (Figure 3A) with weak evidence. This means that when the results of the viscoelastic test were presented with Visual Clot, the anesthesia teams had about a 56% higher rate of correctly performed therapeutic measures. The different scenarios had no significant impact on the team’s performance with respect to this outcome (all *P*>.05).

### Time to Targeted Coagulation Therapy

The median time to administer a first correct targeted coagulation product was 269 (IQR 151-541) seconds when anesthesia teams used Visual Clot, compared with 370 (IQR 207 to upper quartile not reached) seconds when they used ROTEM temograms. Accordingly, the adjusted Cox regression model showed a 158% increase in the probability of administering the first correct targeted coagulation product faster when Visual Clot was used compared with ROTEM temograms (hazard ratio [HR] 2.58, 95% CI 1.37-4.85; *P*=.003; Figure 3B). Concerning this outcome, the teams performed better in the scenarios of aortic arch reconstruction, uterine atony, and kidney transplant than in the scenario of ectopic pregnancy (HR 32.60, 95% CI 9.98-106.43; *P*<.001; HR 5.28, 95% CI 1.82-15.33; *P*=.002; HR 4.02, 95% CI 1.37-11.80; *P*=.01, respectively), independently of the used modality.

### Correct Overall Coagulation Therapy

Concerning the overall coagulation therapy, which also includes actions independent of the interpretation of viscoelastic test results, the Poisson regression model showed no difference between Visual Clot and ROTEM temograms (rate ratio 1.21, 95% CI 0.93-1.58; *P*=.20; Figure 3A). However, the different scenarios impacted team performance independently of the modality used, concerning this outcome. Teams performed better in the aortic arch reconstruction and uterine atony scenarios compared to the ectopic pregnancy scenario (HR 1.76, 95% CI 1.16-2.74; *P*=.01; HR 1.56, 95% CI 1.02-2.45; *P*=.045, respectively). In the scenario of kidney transplant, no significant difference could be shown compared to the scenario of ectopic pregnancy (HR 1.20, 95% CI 0.81-1.84; *P*=.37).

### Perceived Confidence

The mixed logistic regression model revealed more than three and a half times higher odds (odds ratio 3.60, 95% CI 1.49-8.71; *P*=.005) of being confident when Visual Clot was used instead of ROTEM temograms (Figure 3C).

### Perceived Workload

Participants perceived workload burden was similarly distributed between Visual Clot (median 63, IQR 57-70) and ROTEM temograms (median 65, IQR 56-72). Accordingly, the comparison of the 2 technologies in terms of workload using a mixed linear regression model revealed no difference (coefficient –0.03, 95% CI –3.08 to 2.88; *P*=.99; Figure 3D).

### General Statements About Visual Clot

Most participants (64/85, 75% agreed or strongly agreed) who used Visual Clot in the simulation felt better prepared to interpret the results of the viscoelastic test compared to ROTEM temograms. They also agreed or strongly agreed with the statement that Visual Clot is easy to learn (79/85, 93%) and that they would use this technique in clinical practice (73/85, 86%). Most participants (64/85, 75%) disagreed or strongly disagreed with the statement, “Visual Clot seems too simplistic to be any good.” Table 3 shows the exact wording of the 4 statements and the numbers and percentages per statement.

**Table 3:** The participant’s ratings concerning the 4 general statements about Visual Clot (N=85).

General statement about Visual Clot	Strongly agree, n (%)	Agree, n (%)	Disagree, n (%)	Strongly disagree, n (%)
With Visual Clot, I felt better prepared to interpret the result of a viscoelastic test.	21 (25)	43 (51)	18 (21)	3 (3)
The interpretation of Visual Clot was easy to learn.	32 (38)	47 (55)	6 (7)	0 (0)
I would use Visual Clot in everyday clinical practice.	28 (33)	45 (53)	9 (11)	3 (3)
Visual Clot seems too simplistic to be any good.	3 (3)	18 (21)	53 (63)	11 (13)

## Discussion

### Overview

This is the first study to examine Visual Clot in a high-fidelity simulation setting. Visual Clot translates the numerical results of point-of-care viscoelastic testing into an easy-to-interpret animation of a blood clot. We analyzed 59 simulated critical bleeding scenarios performed by 59 anesthesia teams. The teams had either Visual Clot or corresponding ROTEM temograms available to perform targeted coagulation therapy, according to the study center’s guidelines. Teams using Visual Clot were more likely to perform targeted coagulation therapies correctly and faster than teams using ROTEM temograms. Furthermore, team members experienced higher decision confidence through using Visual Clot.

### Principal Findings

Teams using Visual Clot not only performed the first targeted therapy intervention earlier but were also 56% more likely to perform all required targeted therapeutic interventions, indicating a slight superiority, albeit with weak evidence (*P*=.05), of Visual Clot compared to ROTEM temograms. Not yielding a distinct statistical result could be explained by the previous fixed number of participants during the simulation, not allowing for a power analysis.

### Comparison to Previous Work

The indications for improvement in coagulation management by Visual Clot may be explained based on previous research on information transfer in clot-based visualization of viscoelastic test results [7,9,10]. Considering user-centered and situation-awareness design aspects [24], Visual Clot presents numeric viscoelastic test results in a preprocessed form by assigning them into easy-to-understand visualizations with distinct differentiable states without taking the final decision from the user. As a result, users may better understand the interactions and correlations of hemostasis and can make informed treatment decisions for their patients faster and more accurately. For example, it is evident to anyone that an extra heparin icon in an otherwise normal clot formation represents a heparin effect. In order to gather the same information, several ROTEM temograms must be viewed and interpreted simultaneously. Briefly, ROTEM temograms used in the simulations were EXTEM (tests the extrinsic pathway), INTEM (tests the intrinsic pathway), FIBTEM (tests the fibrinogen function), and HEPTEM (tests the heparin effect). Once the EXTEM channel is determined as normal with an INTEM channel indicating a prolonged clotting time, the FIBTEM and HEPTEM channels must also be assessed, as only a normal clotting time in the HEPTEM channel reveals a heparin effect (Figure 2).

Arriaga et al [25] performed a simulation study evaluating a quality and safety intervention, reporting that adherence to essential therapeutic tasks increased by 17% when a surgical crisis checklist was used. When we see these results in the context of our finding that Visual Clot increases adherence to targeted coagulation management by 56%, the positive impact of user-centered result presentation becomes apparent.

Concerning the outcome of overall coagulation therapy, we could not detect a significant difference between Visual Clot and ROTEM temograms. However, this is not surprising since this outcome also includes measures independent of the interpretation of the viscoelastic test results, which we did not influence by our intervention.

Interestingly, previous computer-based studies found a significant reduction in perceived workload when anesthesiologists and intensivists used Visual Clot instead of ROTEM temograms to interpret the viscoelastic test [7,9]. We did not find this workload reduction in our high-fidelity simulation. This could be attributed to the previous studies solely investigating the interpretation of the viscoelastic test result and not subjecting participants to other stressors, such as maintenance of anesthesia and hemodynamics, fluid management, and manual tasks (such as drawing blood, putting in venous lines, or endotracheal intubation), as was the case in this simulation study. It will be interesting to see whether this result translates into the everyday clinical setting in future Visual Clot studies and provokes thoughts on how to further optimize this novel tool.

### Limitations and Strengths

This study has limitations. First, simulation fidelity and authenticity are inherent limitations in all simulation-based research. Simulation may not reflect a real critical bleeding emergency’s psychological and temporal pressure and dynamics. However, putting the simulation bias into perspective, Merry et al [11] showed that randomized simulation trials allow the same conclusions as clinical randomized controlled trials. Furthermore, a high-fidelity simulation provides an established evaluation tool in medicine and enables us to test Visual Clot in emergency bleeding scenarios safely before its use in real clinical practice [11,26]. The study design and efforts to replicate clinical reality as closely as possible using a state-of-the-art patient simulator, actors portraying the surgeon, real medications, and airway equipment guaranteed our participants an experience that closely resembled daily clinical practice. Nearly 157 (88%) of the 178 participants valued our simulation as realistic, complementing the study design and execution. Finally, we used video analysis to meet the highest standard in evaluating simulation research [27,28]. Second, this was a single-center study in a tertiary care hospital in central Europe, with participants accustomed to simulation training and point-of-care viscoelastic testing-guided coagulation management. The study results may vary under different circumstances. Some participants had little to no previous ROTEM experience, whereas others considered themselves well-versed, representing a broad spectrum from beginners to advanced medical staff in terms of viscoelastic testing. Third, when analyzing these results, we treat teams as independent, even though an individual may be a member of another team. Fourth, we did not perform a pilot study or sample size calculation because the simulation study was embedded in the annual simulation training of the Institute of Anesthesiology, University Hospital Zurich, Switzerland. It should be noted that common ethical and economic arguments for a sample size calculation are not applicable to this study because of the given number of simulation days, the number of simulations, and the personnel available during this period [29,30]. However, scientific arguments are valid because this study seems underpowered. With a larger sample size, we may have achieved a clearer difference in the outcome of correct total coagulation therapy favoring Visual Clot [29,30]. Fifth, we generated and used Visual Clot animations and corresponding ROTEM temograms that were clearly attributable to coagulopathy. The viscoelastic results in real clinical bleeding cases may be more elusive. However, this bias applies to both interventions tested and should therefore not play a role in the comparison of the 2 results presented. Future studies are needed to confirm the results of this study by using a prototype that analyzes and displays real blood samples of patients with coagulopathies.

### Conclusion

The use of Visual Clot, compared with ROTEM temograms, resulted in faster administration of the first targeted coagulation product, and the overall targeted coagulation therapy was more likely to be delivered correctly, resulting in high decision confidence and excellent user acceptance. Considering these results of Visual Clot, it could be interesting to suggest all relevant viscoelastic test manufacturers consider augmenting their complex result presentation with a user-centered, intuitive, and easy-to-understand visualization to ease the burden on users deriving from unnecessary cognitive load and to enhance patient care.

## Appendix

Multimedia Appendix 1 Coagulation management algorithm.

Multimedia Appendix 2 Instructional Visual Clot Video.

Multimedia Appendix 3 Case description, team briefing and viscoelastic test result presentation per scenario.

Multimedia Appendix 4 Overview of simulation room.

Multimedia Appendix 5 General Statements about Visual Clot Questionnaire.

Multimedia Appendix 6 Example video sequence which was analyzed.

Multimedia Appendix 7 Simulation-Based Research Extensions for the CONSORT and STROBE Statement Checklist.

## Abbreviations

CONSORT: Consolidated Standards of Reporting Trials
HR: hazard ratio
NASA TLX: National Aeronautics and Space Administration Task Load Index
ROTEM: rotational thrombelastometry
STROBE: Strengthening the Reporting of Observational Studies in Epidemiology
TEG: thromboelastography

## Data Availability

The data sets generated during and/or analyzed during this study are available from the corresponding author on reasonable request.
