# Supplementary material for: Coagulation Management of Critically Bleeding Patients With Viscoelastic Testing Presented as a 3D-Animated Blood Clot (The Visual Clot): Randomized Controlled High-Fidelity Simulation Study
Source: J Med Internet Res. 2023 Oct 12;25:e43895. doi: 10.2196/43895 (PMC10603564; doi:10.2196/43895)

**Multimedia Appendix 1**

Coagulation management algorithm Institute of Anesthesiology, University Hospital, Zurich, Switzerland.


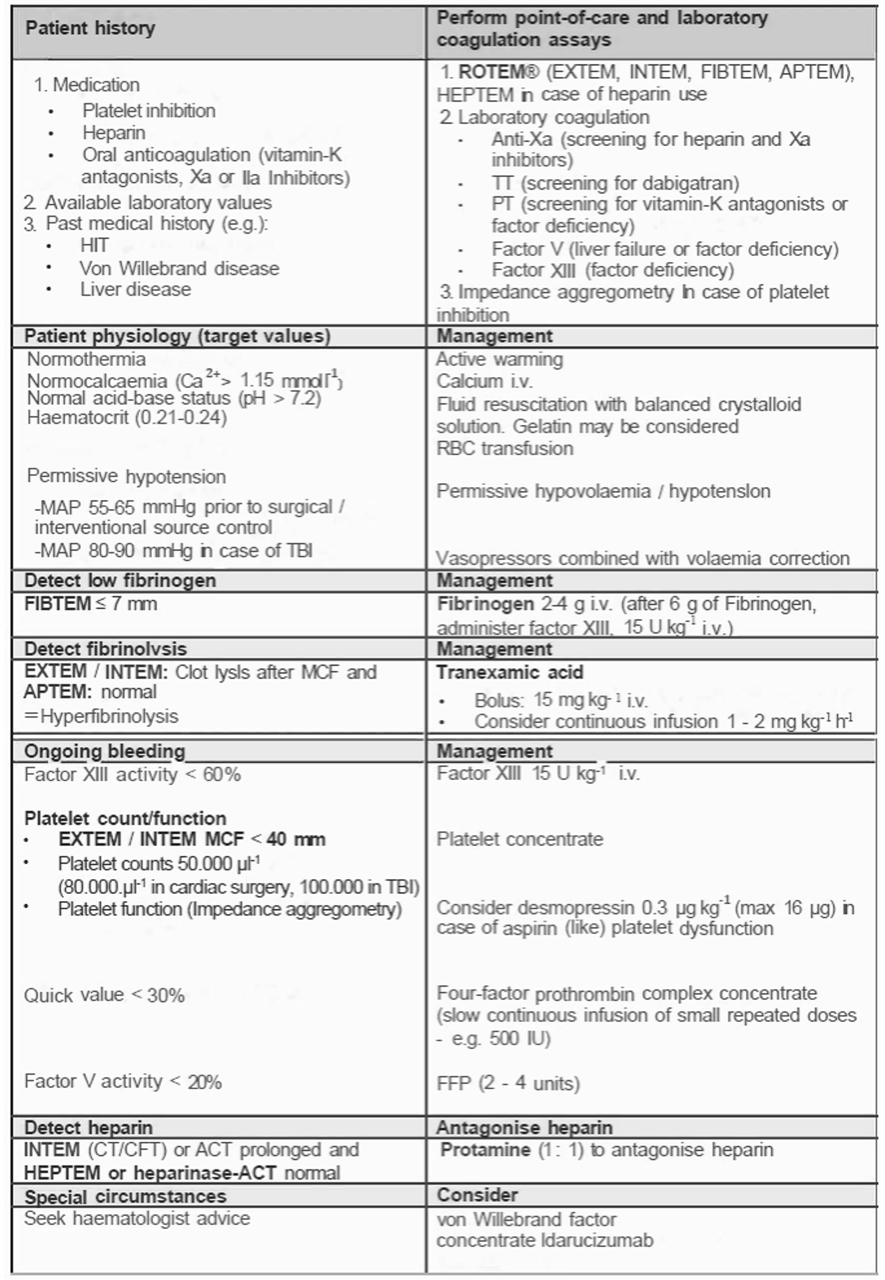

Supplement: Multimedia Appendix 1 [file jmir_v25i1e43895_app1.docx]
