# Supplementary material for: Coagulation Management of Critically Bleeding Patients With Viscoelastic Testing Presented as a 3D-Animated Blood Clot (The Visual Clot): Randomized Controlled High-Fidelity Simulation Study
Source: J Med Internet Res. 2023 Oct 12;25:e43895. doi: 10.2196/43895 (PMC10603564; doi:10.2196/43895)
Supplement: Multimedia Appendix 3 [file jmir_v25i1e43895_app3.docx]

Multimedia Appendix 3

**Improved coagulation management of critically bleeding patients with animated viscoelastic test results - The Visual Clot: A High-Fidelity Simulation**

**Table S1.** General information.

| General information | | Available material (for all scenarios): monitor, anaesthesia cart, airway trolley, anaesthesia ventilator, drip stand, multi-channel infusion pump stand, suction device, patient warming system, Level 1® fast fluid warmer, defibrillator.  Initial laboratory and viscoelastic test results (either as ROTEM temograms or Visual Clot) are presented after five minutes into the scenario (regardless if these were requested). A follow-up laboratory result is available and requires the participants to draw blood from the patient. | | |
| --- | --- | --- | --- | --- |
| **Table S2.** Overview scenario 1: Ectopic pregnancy. | | | | |
| **Scenario 1. Ectopic Pregnancy** | | | | |
| Short scenario description | | A 34 year old, female patient is planned for an urgent laparoscopy due to an ectopic pregnancy with free pelvic fluid. The scenario starts with preparation for anaesthesia induction. The patient is very worried about the outcome of her first pregnancy. The peripheral venous catheter needs to be replaced due to tenderness and shortly after the patient is monitored her blood pressure drops. In addition, the surgeon puts pressure on the team to induce anaesthesia promptly. The goal is to stabilize the patient and correctly administer coagulation treatment based on laboratory and viscoelastic test results. | | |
| Participant briefing | | Ms Bear, 34 year old patient, is currently eight weeks pregnant with her first child. She went to the emergency room (E.R.) this morning due to severe abdominal pain. The transvaginal ultrasound showed an ectopic corneal-interstitial implantation with free pelvic fluid. She weighs 70kg, has no pre-existing medical illnesses and is allergic to an unknown antibiotic and pollen, her last meal was last night. She received one gram of Metamizol in the E.R., blood samples were taken (including blood gas analysis and a viscoelastic test) which are currently being processed. The patient is very worried and moves a lot.  The scenario starts with preparation for anaesthesia induction for an urgent laparoscopic exploration.  *Existing installations:* peripheral venous catheter. | | |
| Laboratory results | | *Initial:* Haemoglobin 89 g.dl-1, Haematocrit 0.28 g.l-1, pH 7.36, Base Excess 3 mmol.l-1, Bicarbonate 21 mmol.l-1, Calcium ionised 1 mmol.l-1, Quick 75%, aPPT 29 seconds, Factor XIII 70%, Factor V 85%.  *Follow-up:* Haemoglobin 91 g.dl-1, Haematocrit 0.3 g.l-1, pH 7.37, Base Excess -2.6 mmol.l-1, Bicarbonate 22 mmol.l-1, Calcium ionised 1 mmol.l-1 | | |
| Visual Clot | | *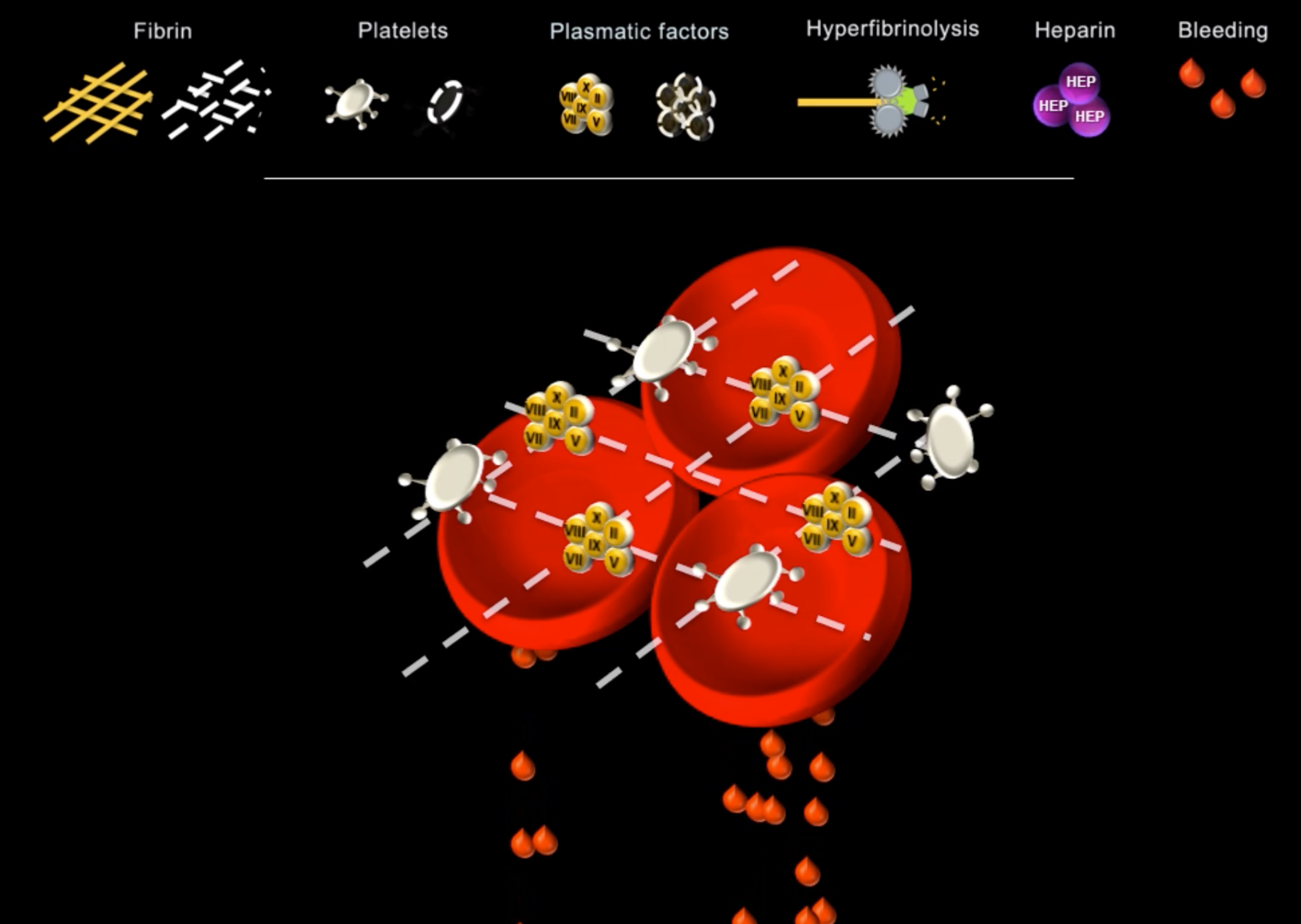* | | |
| ROTEM temograms | | *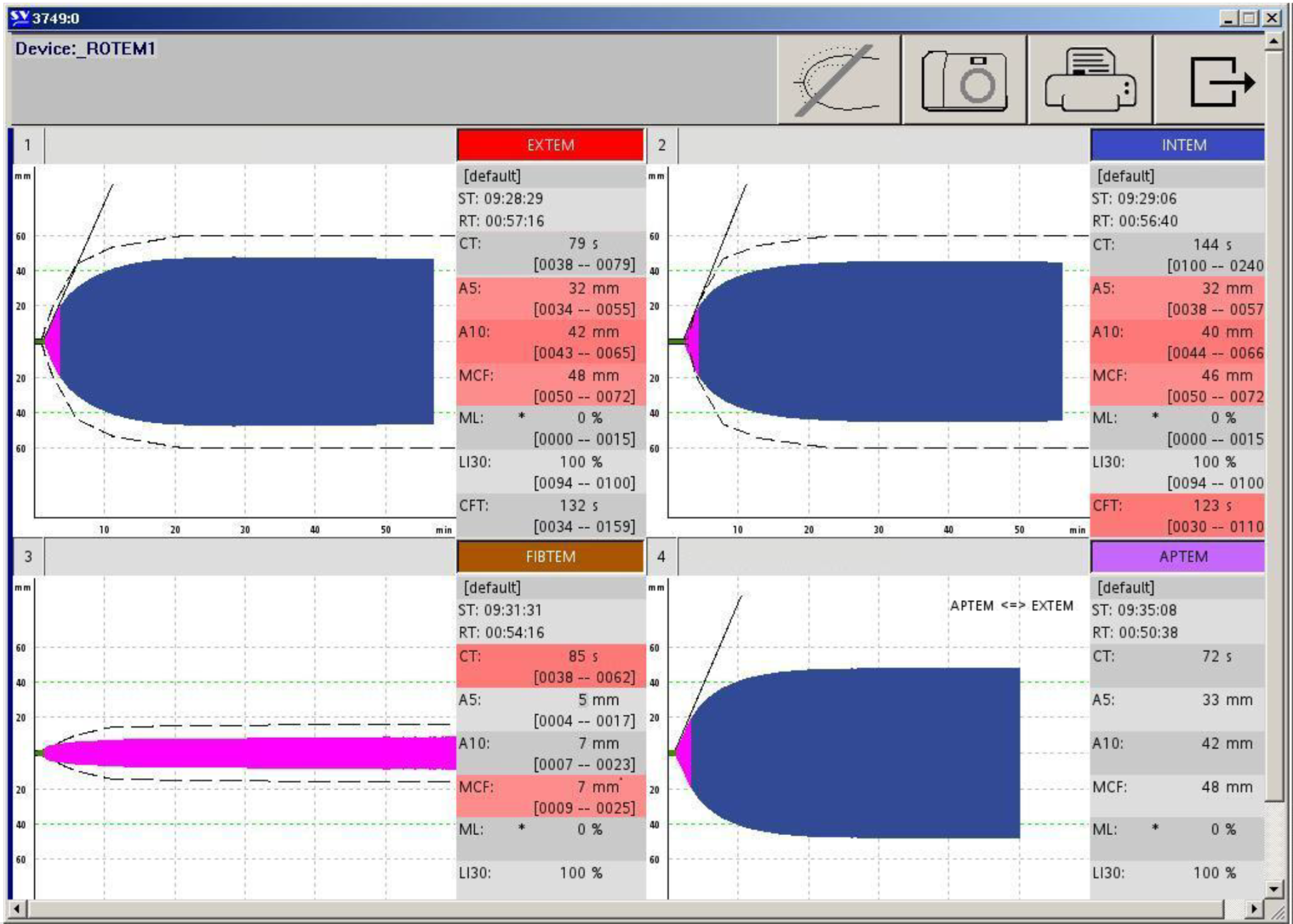* | | |
| Detailed scenario description | | | | |
| Time (minutes) | Vital signs | Events | Surgeon (S) /  Patient (P) | Tasks to be performed |
| 0 - 1 | Patient not monitored, no vital signs available | Patient arrived in the operating room for anaesthesia induction | (P) Mentions pain on left arm | (1) Start installing monitoring and check peripheral venous catheter |
| 1 - 3 | HR: 96min-1  BP: 127/76mmHg  S_p_O_2_: 96%  Temp: 35.3°C | Peripheral venous catheter not working and painful | (S) Asks how long induction will take and to hurry up  (P) Is very worried about the procedure and outcome | (2) Install new peripheral venous catheter |
| 3 - 5 | HR: 110min-1  BP: 80/45mmHg  S_p_O_2_:96%  Temp: 35.3°C | Sudden drop of blood pressure due to increased free pelvic fluid | (P) Expresses feeling light headedness | (3) Place patient in Trendelenburg position  (4) Apply fluid bolus (blood pressure increases after >1.5 litres)  (5) consider ephedrine  (6) call senior physician |
| 5-7 | HR: 118min-1  BP: 87/45mmHg  S_p_O_2_: 94%  Temp: 35.3°C | Viscoelastic test and laboratory results arrive | (S) Urges to start surgery promptly | (7) 10 for 10, discuss results  (8) Start coagulation management: tranexamic acid, calcium, fibrinogen, fluids and warming |
| 7-9 | HR: 115min-1  BP: 89/46mmHg  S_p_O_2_: 95%  Temp: 35.3°C | “ | “ | (9) continue with coagulation management |
| 9-11 | HR: 108min-1  BP: 95/74mmHg  S_p_O_2_: 95%  Temp: 35.3°C | Patient shows signs of decreased vigilance | (P) only responds with inappropriate words or inconprehensible sounds | “ |
| 11-13 | HR: 101min-1  BP: 79/43mmHg  S_p_O_2_: 97%  Temp: 35.6°C | “ | “ | “ |
| 13-15 | HR: 92min-1  BP: 123/79mmHg  S_p_O_2_: 98%  Temp: 35.9°C | Patient becomes stable | (P) feels better | (10) Proceed with induction (uneventful) |

**Table S3.** Overview scenario 2: Aortic arch reconstruction.

| **Scenario 2. Aortic Arch Reconstruction** | | | | |
| --- | --- | --- | --- | --- |
| Short scenario description | | After an uneventful aortic arch reconstruction on a 74 year old, male patient, the surgeon notes diffuse bleeding whilst stitching up. The patient was weaned off the cardiopulmonary bypass machine twenty minutes prior. The surgeons cannot find a source of the bleeding and blame poor coagulation management. The patient deteriorates haemodynamically until coagulation is corrected. The goal is to stabilize the patient and correctly administer coagulation treatment based on laboratory and viscoelastic test results. | | |
| Participant briefing | | Mr Bogner, 74 years old, is currently undergoing an aortic arch reconstruction in the operating room. He weighs 80kg, has no allergies and received three coronary stents five years ago. He was weaned off the cardiopulmonary bypass machine twenty minutes earlier, the viscoelastic test result prior presented normal findings. The perfusionist was content with the activated clotting time. Heparin was antagonised with protamine in a 1:1 plus 10'000 IE ratio. He received 1g of tranexamic acid bolus during anaesthesia induction with a respective infusion pump running since at 10mg.kg-1.hour-1. The surgery was uneventful, the patient was stable throughout. The surgeon has been stitching up for 15 minutes.  *Ongoing infusion pumps:* tranexamic acid 800mg.h-1, norepinephrine 6µg.min-1, sufentanyl 30µg.h-1  *Existing installations:* peripheral venous catheter, radial artery catheter, central venous catheter, pulmonary artery catheter, urinary catheter, tracheal tube  *Other material:* picture of an open thorax after aortic arch reconstruction on the manekin. | | |
| Laboratory results | | *Initial*: Haemoglobin 90 g.dl-1, Haematocrit 0.26 g.l-1, pH 7.38, Base Excess -1.5 mmol.l-1, Bicarbonate 23 mmol.l-1, Calcium ionised 0.85 mmol.l-1, Quick 45%, aPPT 76 seconds, factor XIII 70%, factor V 35%.  *Follow-up*: Haemoglobin 89 g.dl-1, Haematocrit 0.25 g.l-1, pH 7.37, Base Excess -1.5 mmol.l-1, Bicarbonate 23.1 mmol.l-1, Calcium ionised 0.88 mmol.l-1 | | |
| Visual Clot | | *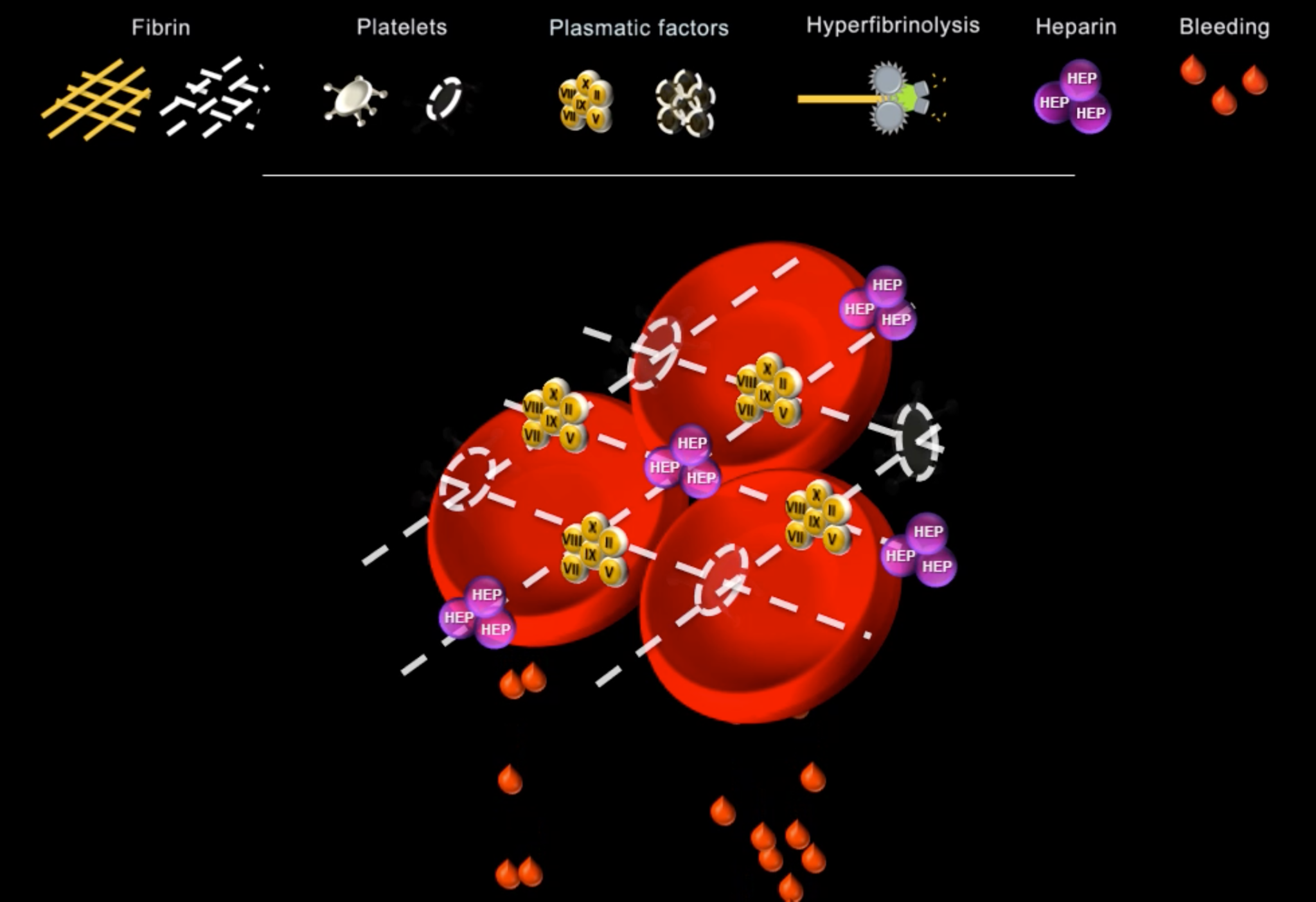* | | |
| ROTEM temograms | | *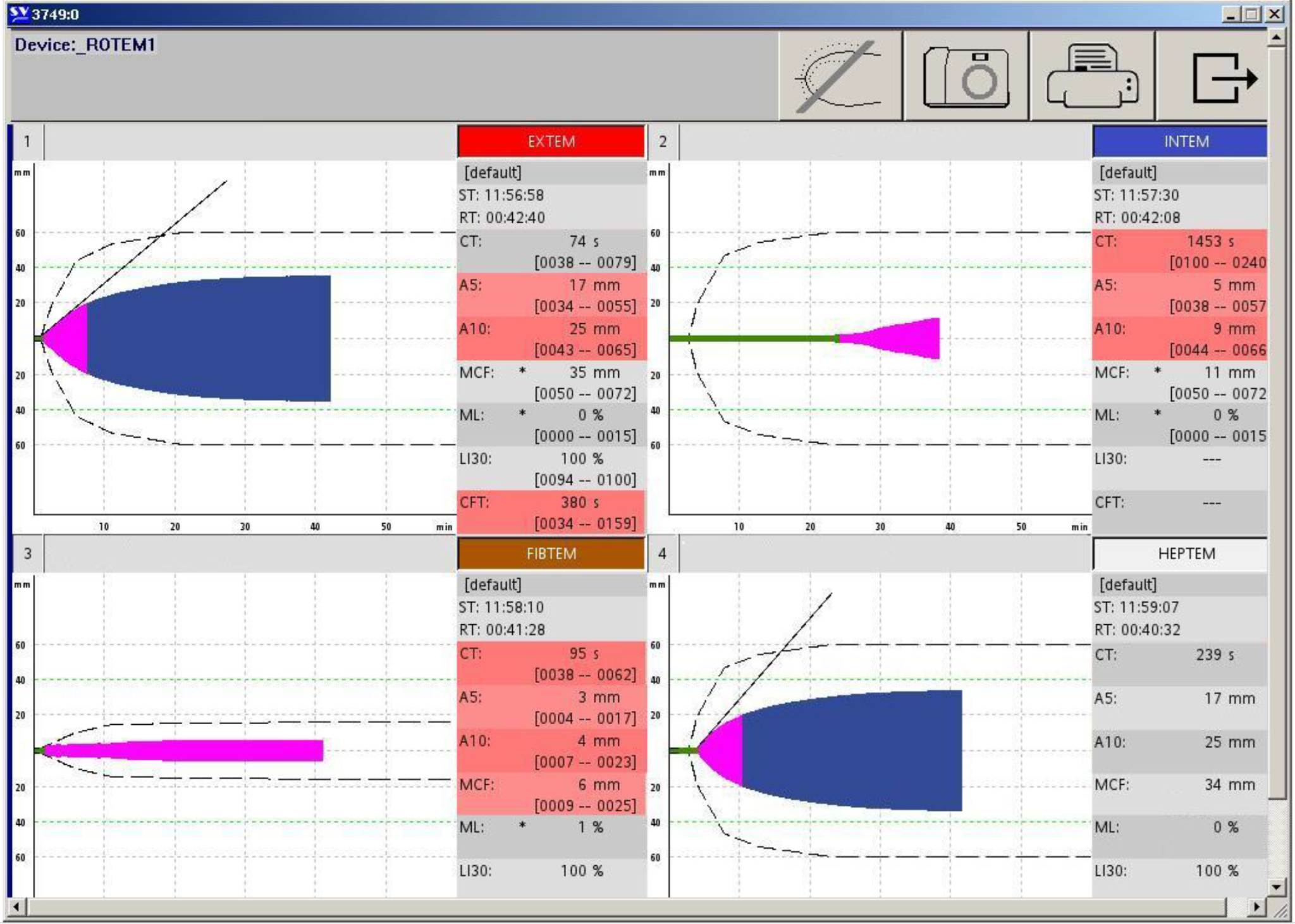* | | |
| Detailed scenario description | | | | |
| Time (minutes) | Vital signs | Events | Surgeon | Tasks to be performed |
| 0 - 1 | HR: 80min-1  BP: 130/85mmHg  S_p_O_2_: 98%  Temp: 35.8°C | Patient was weaned off cardiopulmonary bypass machine, heparin was reversed with protamine as per standard, previous lab test unremarkable | Is stitching up and states the surgery went very well |  |
| 1 - 3 | HR: 89min-1  BP: 125/73mmHg  S_p_O_2_: 98%  Temp: 35.8°C | Diffuse bleeding begins | Notes diffuse bleeding, is annoyed and blames poor coagulation management | (1) Check surgery site and surgical suction bag |
| 3 - 5 | HR: 105min-1  BP: 109/61mmHg  S_p_O_2_: 98%  Temp: 35.8°C | Surgeon needs to suction more. Noticeable change in haemodynamics | Complains further, cannot identify a source of bleeding | (2) Increase norepinephrine  (3) Draw arterial blood gas analysis and ROTEM  (4) Call senior physician |
| 5 - 7 | HR: 111min-1  BP: 100/57mmHg  S_p_O_2_: 96%  Temp: 35.8°C | Viscoelastic test and laboratory results arrive | “ | (5) Further increase norepinephrine  (6) Start coagulation management: protamine, platelets, fibrinogen, calcium, and warming |
| 7 - 9 | HR: 115min-1  BP: 94/55mmHg  S_p_O_2_: 98%  Temp: 36.0°C | Diffuse bleeding continues | Mentions no improvement in coagulation | (7) continue with coagulation management |
| 9 - 11 | HR: 113min-1  BP: 92/50mmHg  S_p_O_2_: 98%  Temp: 36.2°C | “ | Asks what measures were taken | “ |
| 11 - 13 | HR: 108min-1  BP: 102/58mmHg  S_p_O_2_: 98%  Temp: 36.2°C | “ | “ | “ |
| 13 - 15 | HR: 104min-1  BP: 115/68mmHg  S_p_O_2_: 98%  Temp: 36.4°C | Patient becomes stable | Bleeding has stopped, will finish surgery shortly | (8) proceed with anaesthesia |

**Table S4.** Overview scenario 3: Uterine atony.

| **Scenario 3. Uterine atony** | | | | |
| --- | --- | --- | --- | --- |
| Short scenario description | | A 35 year old woman with a twin pregnancy is currently undergoing planned caesarean section under general anaesthesia due to HELLP (Hemolysis, Elevated Liver enzymes and Low Platelets) syndrome with deterioration of laboratory parameters. The twins have already been delivered and are taken care for by neonatology. The scenario begins with the obstetrician mobilising the placentas, which will lead to uterine atony with severe post-partum haemorrhage. The goal is to stabilize the patient and correctly administer coagulation treatment based on laboratory and viscoelastic test results. | | |
| Participant briefing | | A 35 year old female patient is in the operating room for a planned caesarean section for a twin pregnancy. The pregnancy was uneventful, though the patient was hospitalised three days prior due to HELLP syndrome. Fetal lung maturity is completed. Laboratory values, with focus on platelets, are progressively worsening and therefor she receives general anaesthesia. The patient does not have any allergies and a normal body weight. At the beginning of the scenario the twins are already in neonatal care.  *Ongoing infusion pumps:* neosynephrine 6 ml.h-1, sufentanyl 30 µg.h-1  *Existing installations:* two peripheral venous catheter, radial artery catheter, urinary catheter, tracheal tube.  *Other material:* two placenta replicas for simulation purpose. | | |
| Laboratory results | | *Initial*: Haemoglobin 80 g.dl-1, Haematocrit 0.25 g.l-1, pH 7.42, Base Excess -1.2 mmol.l-1, Bicarbonate 25 mmol.l-1, Calcium ionised 0.90 mmol.l-1, Quick 85%, aPPT 39 seconds, factor XIII 95%, factor V 85%.  *Follow-up*: Haemoglobin 82 g.dl-1, Haematocrit 0.26 g.l-1, pH 7.43, Base Excess 0.2 mmol.l-1, Bicarbonate 24 mmol.l-1, Calcium ionised 1.23 mmol.l-1 | | |
| Visual Clot | | *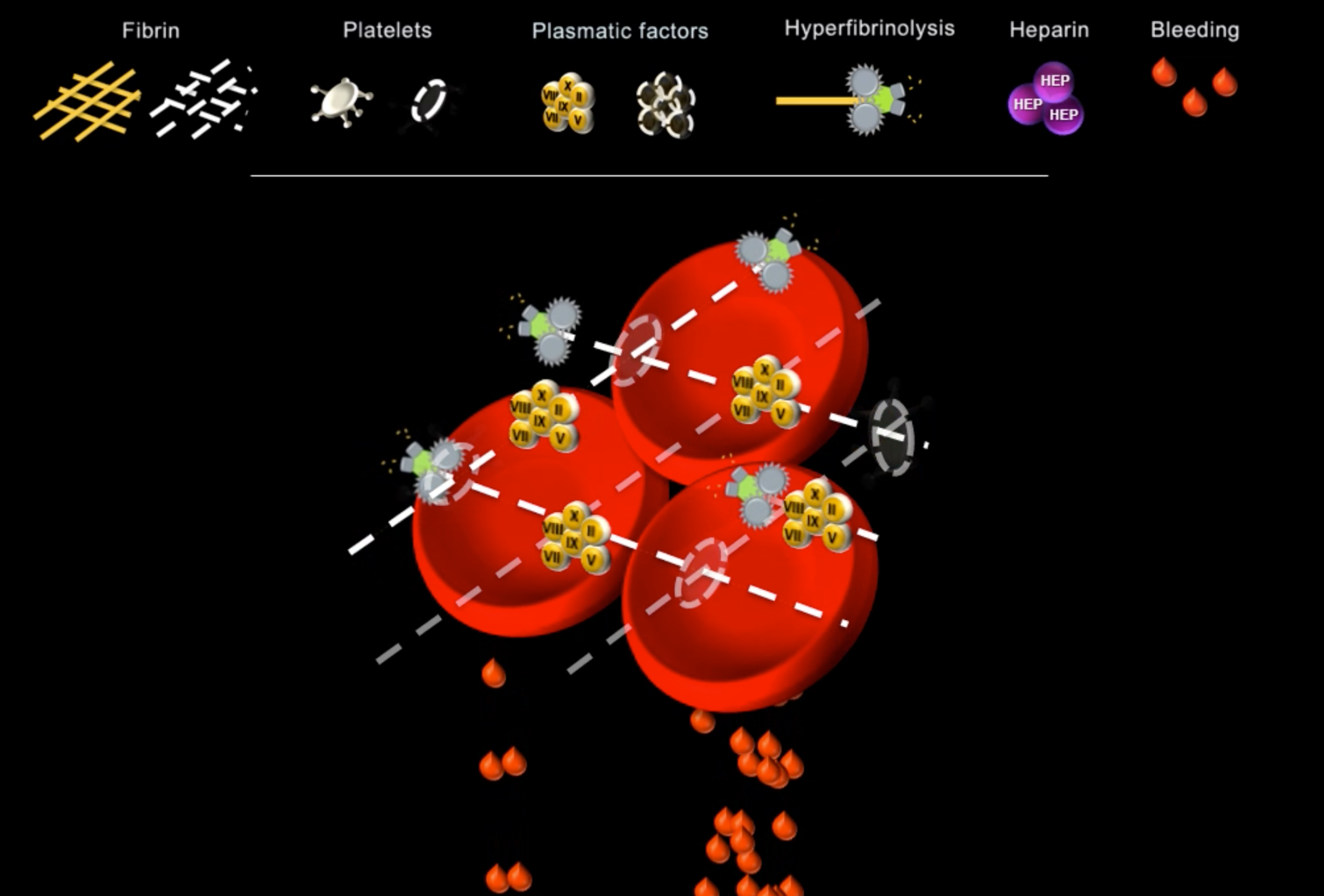* | | |
| ROTEM temograms | | *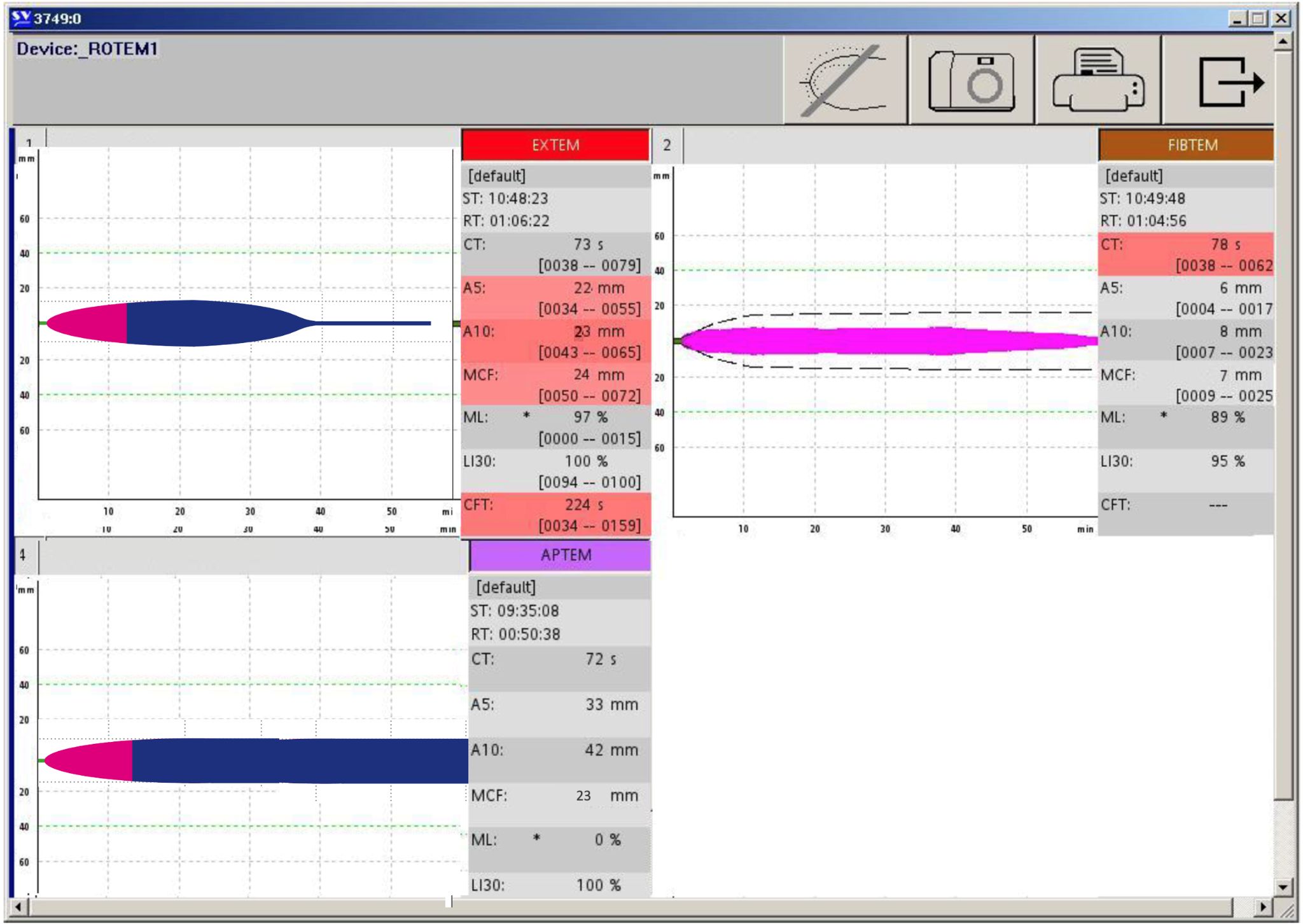* | | |
| Detailed scenario description | | | | |
| Time (minutes) | Vital signs | Events | Surgeon | Tasks to be performed |
| 0 - 1 | HR: 90min-1  BP: 120/65mmHg  S_p_O_2_: 98%  Temp: 36.4°C | Patient fully monitored, intubated, stable vital signs | Obstetrician is relaxed, twins were delivered, is now mobilising placentas |  |
| 1 - 3 | HR: 90min-1  BP: 120/65mmHg  S_p_O_2_: 96%  Temp: 36.4°C | Placentas are being mobilised | Asks to administer ceftriaxone and 5 IU oxytocin | (1) Draw up and administer drugs |
| 3 - 5 | HR: 98min-1  BP: 100/80mmHg  S_p_O_2_: 96%  Temp: 36.2°C | Both placentas have been removed when a uterine atony is communicated. Drop in blood pressure | States the second placenta is out, the uterus feels soft and fills with blood. Asks to give additional 40 IU oxytocin to the infusion | (2) Administer oxytocin and fluid bolus  (3) Draw bloods  (4) Call senior physician |
| 5 - 7 | HR: 110min-1  BP: 92/60mmHg  S_p_O_2_: 94%  Temp: 36.0°C | Blood loss due to post-partum haemorrhage  Viscoelastic test and laboratory results arrive | Needs to suction a lot of blood and notes no change in tone of uterus, despite compression | (5) Increase neosynephrine  (6) Start coagulation management: platelets, fibrinogen, calcium, tranexamic acid and warming |
| 7 - 9 | HR: 118min-1  BP: 89/46mmHg  S_p_O_2_: 95%  Temp: 35.7°C | “ | Inquires about the patient's vital signs | (7) Proceed with coagulation management |
| 9 - 11 | HR: 108min-1  BP: 95/74mmHg  S_p_O_2_: 95%  Temp: 36.0°C | Bleeding continues | Suggests treatment with sulprostone, if not already applied | (8) Call midwife to prepare sulprostone and administer accordingly  (9) continue with coagulation management |
| 11 - 13 | HR: 100min-1  BP: 79/43mmHg  S_p_O_2_: 97%  Temp: 36.1°C | “ | Inquires about the patient's well being | “ |
| 13 - 15 | HR: 93min-1  BP: 123/79mmHg  S_p_O_2_: 98%  Temp: 36.3°C | Uterus regains normal tone, bleeding stops | Bleeding has stopped, will proceed with stitching up | (10) Proceed with anaesthesia |

**Table S5.** Overview scenario 4: Kidney transplant.

| **Scenario 4. Kidney transplant** | | | | |
| --- | --- | --- | --- | --- |
| Short scenario description | | The anaesthesia team is providing for a patient during a kidney transplant surgery. Induction was uneventful followed by a regular course of surgery, despite difficult preparation of the kidney. Shortly before reperfusion, the surgical field fills with blood, the surgeon shows difficulties in clamping a ruptured vessel. If the team does not take corrective action, the patient falls into cardiac arrest. The goal is to stabilize the patient and correctly administer coagulation treatment based on laboratory and viscoelastic test results. | | |
| Participant briefing | | A 36 year old female dialysis patient, weighing 65kg, with autosomal dominant polycystic kidney disease is undergoing a kidney transplant. The patient has no other underlying conditions, does not take any anticoagulants and has no allergies. Anaesthesia induction was uneventful and despite difficult preparation of the kidney, the surgery went according to plan. The scenario begins shortly before reperfusion of the kidney.  *Ongoing infusion pumps:* norepinephrine 3µg.min-1  *Existing installations:* two peripheral venous catheter, radial artery catheter, central venous catheter, urinary catheter, tracheal tube.  *Other material:* picture of the surgical site of a kidney transplant on the manikin. | | |
| Laboratory results | | *Initial*: Haemoglobin 50 g.dl-1, Haematocrit 0.15 g.l-1, pH 7.10, Base Excess 10 mmol.l-1, Bicarbonate 15 mmol.l-1, Calcium ionised 0.80 mmol.l-1, Quick 25%, aPPT 60 seconds, factor XIII 30%, factor V 15%.  *Follow-up*: Haemoglobin 58 g.dl-1, Haematocrit 0.20 g.l-1, pH 7.2, Base Excess -10 mmol.l-1, Bicarbonate 17 mmol.l-1, Calcium ionised 0.95 mmol.l-1 | | |
| Visual Clot | | *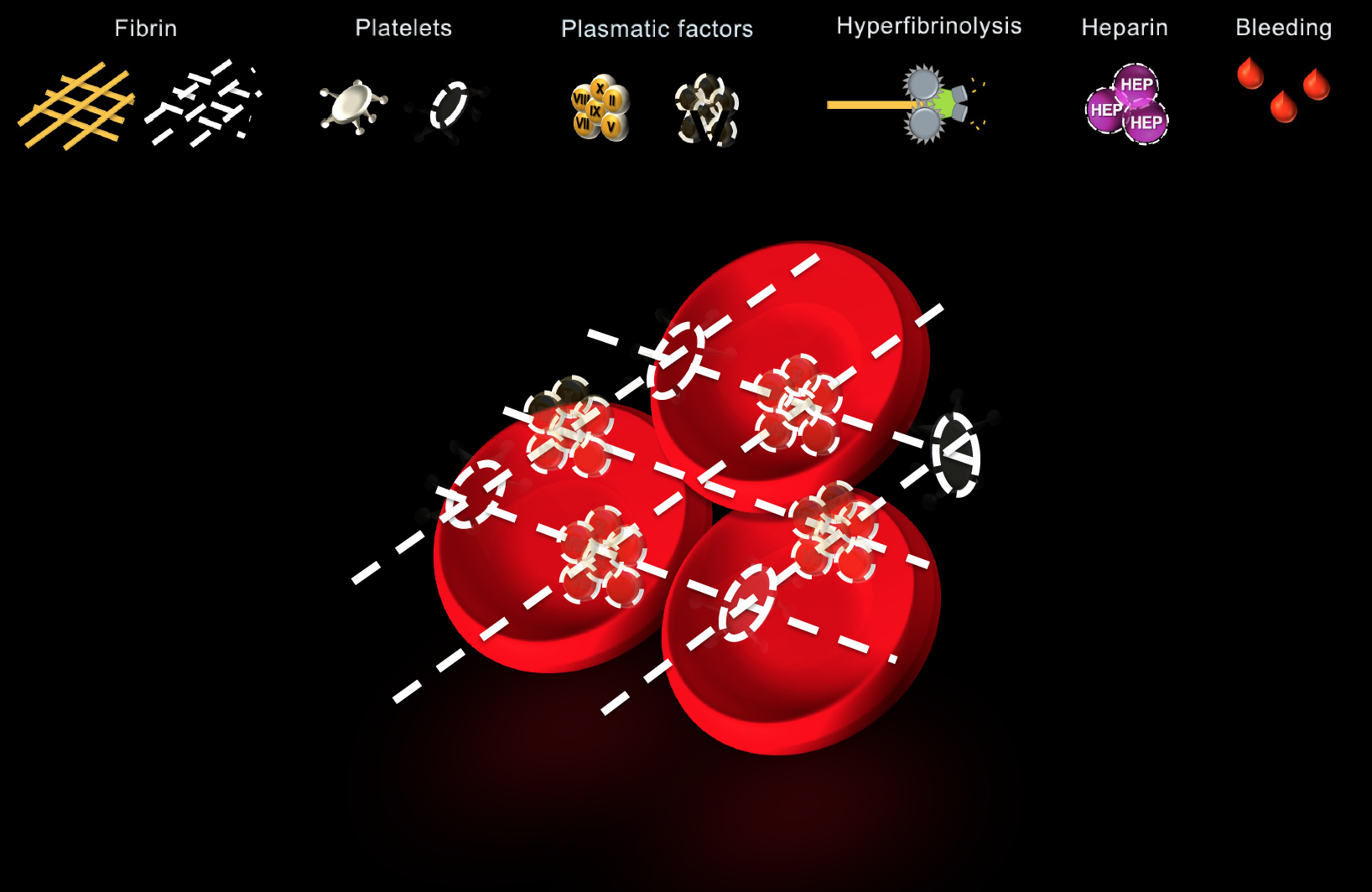* | | |
| ROTEM temograms | | *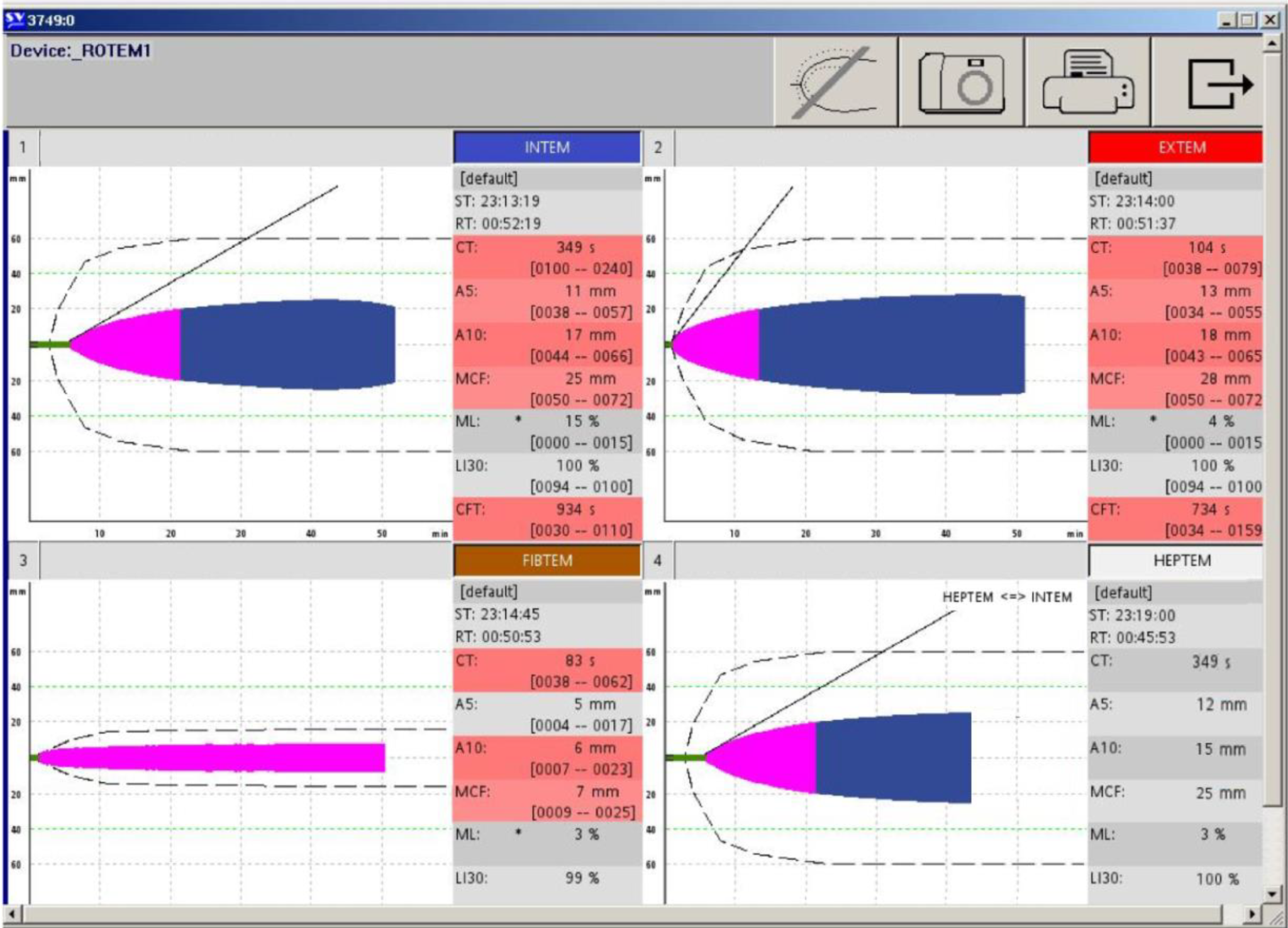* | | |
| Detailed scenario description | | | | |
| Time (minutes) | Vital signs | Events | Surgeon | Tasks to be performed |
| 0 - 1 | HR: 65min-1  BP: 103/90mmHg  S_p_O_2_: 98%  Temp: 36.0°C | Patient fully monitored, intubated, stable vital signs. Prolonged ischemia time stresses surgeons. | Slightly annoyed. States it is going well but urges to proceed with reperfusion shortly |  |
| 1 - 3 | HR: 95min-1  BP: 105/61mmHg  S_p_O_2_: 95%  Temp: 36.0°C | Surgeon damages a vessel | Quietly begins to suction | (1) Administer fluid bolus, increase norepinephrine  (2) Communicate with surgeons if there is a problem |
| 3 - 5 | HR: 129min-1  BP: 86/43mmHg  S_p_O_2_: 94%  Temp: 36.0°C | Bleeding in the surgical site occurs | Calmly communicates that it is bleeding and that clamping the vessel is difficult | (3) Draw blood for ROTEM and arterial blood gas analysis  (4) Set 100% FiO2 and increase fresh gas flow  (5) call senior physician |
| 5 - 7 | HR: 135min-1  BP: 73/43mmHg  S_p_O_2_: 92%  Temp: 35.8°C | Bleeding becomes uncontrolled.  Viscoelastic test and laboratory results arrive | Becomes nervous, says it is bleeding more and more | (6) 10 for 10 with surgeon  (7) Start coagulation management: warming, calcium, packed red blood cells, platelets, fibrinogen, factor XIII, fluids, four factor prothrombin complex, fresh frozen plasma |
| 7 - 9 | HR 138: min-1  BP: 65/41mmHg  S_p_O_2_: 92%  Temp: 35.6°C | “ | Cannot control the bleeding, asks to call for their back-up for help | (8) Proceed with coagulation management  (9) call surgeon back up |
| 9 - 11 | HR: 127min-1  BP: 65/32mmHg  S_p_O_2_: 44%  Temp: 35.3°C | Patient falls into cardiac arrest (pulsless electrical activity) | Quietly tries to control the bleeding | (10) Start CPR according to guidelines, assign chest compressions to second surgeon  (11) continue with coagulation management |
| 11 - 13 | HR: 132min-1  BP: 71/33mmHg  S_p_O_2_: 61%  Temp: 35.3°C | “ | Announces the bleeding stopped and that they were able to clamp the vessel | (12) continue with coagulation management |
| 13 - 15 | HR: 68min-1  BP: 80/50mmHg  S_p_O_2_: 93%  Temp: 35.4°C | Return of spontaneous circulation | Continues with surgery | (13) Proceed with anaesthesia and organise intensive care bed |
