# Supplementary material for: Coagulation Management of Critically Bleeding Patients With Viscoelastic Testing Presented as a 3D-Animated Blood Clot (The Visual Clot): Randomized Controlled High-Fidelity Simulation Study
Source: J Med Internet Res. 2023 Oct 12;25:e43895. doi: 10.2196/43895 (PMC10603564; doi:10.2196/43895)

Figure S1. Simulation Setup. (a) Simulation room resembling an operating theatre with the available equipment. (b) Adjacent room with study investigators and simulator technician.

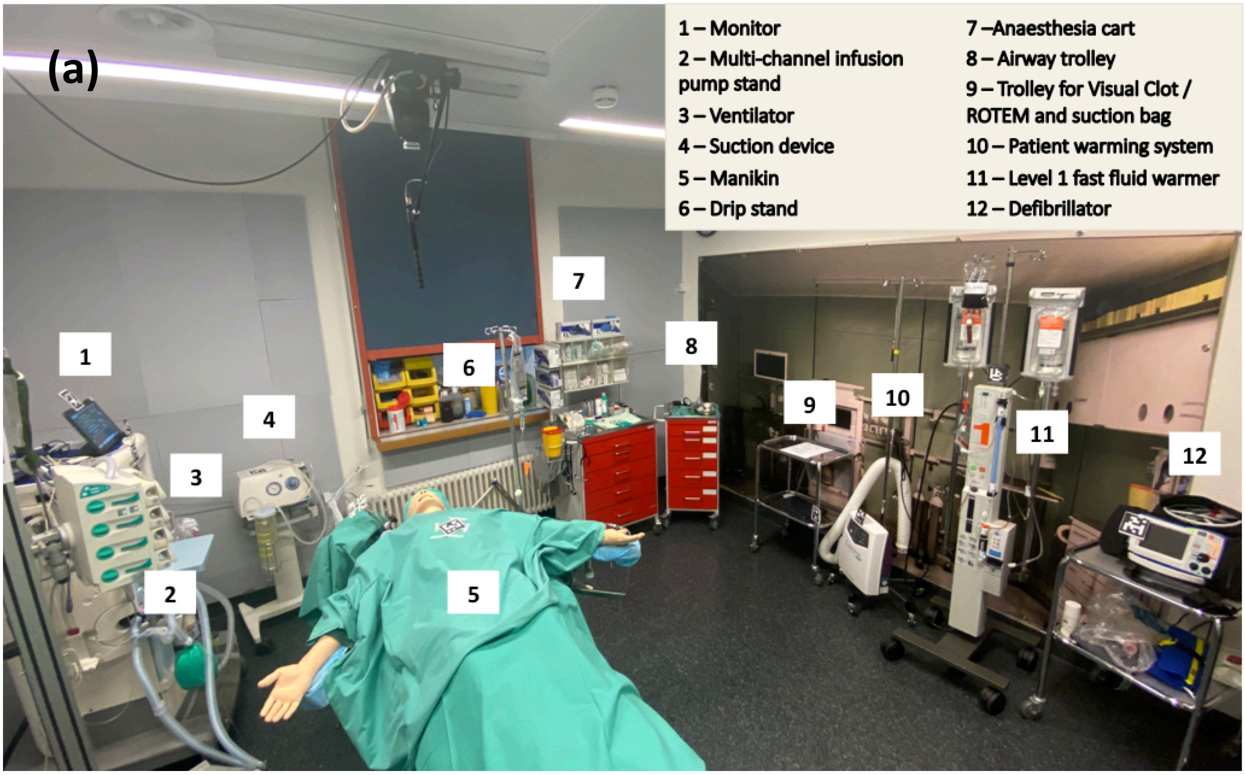

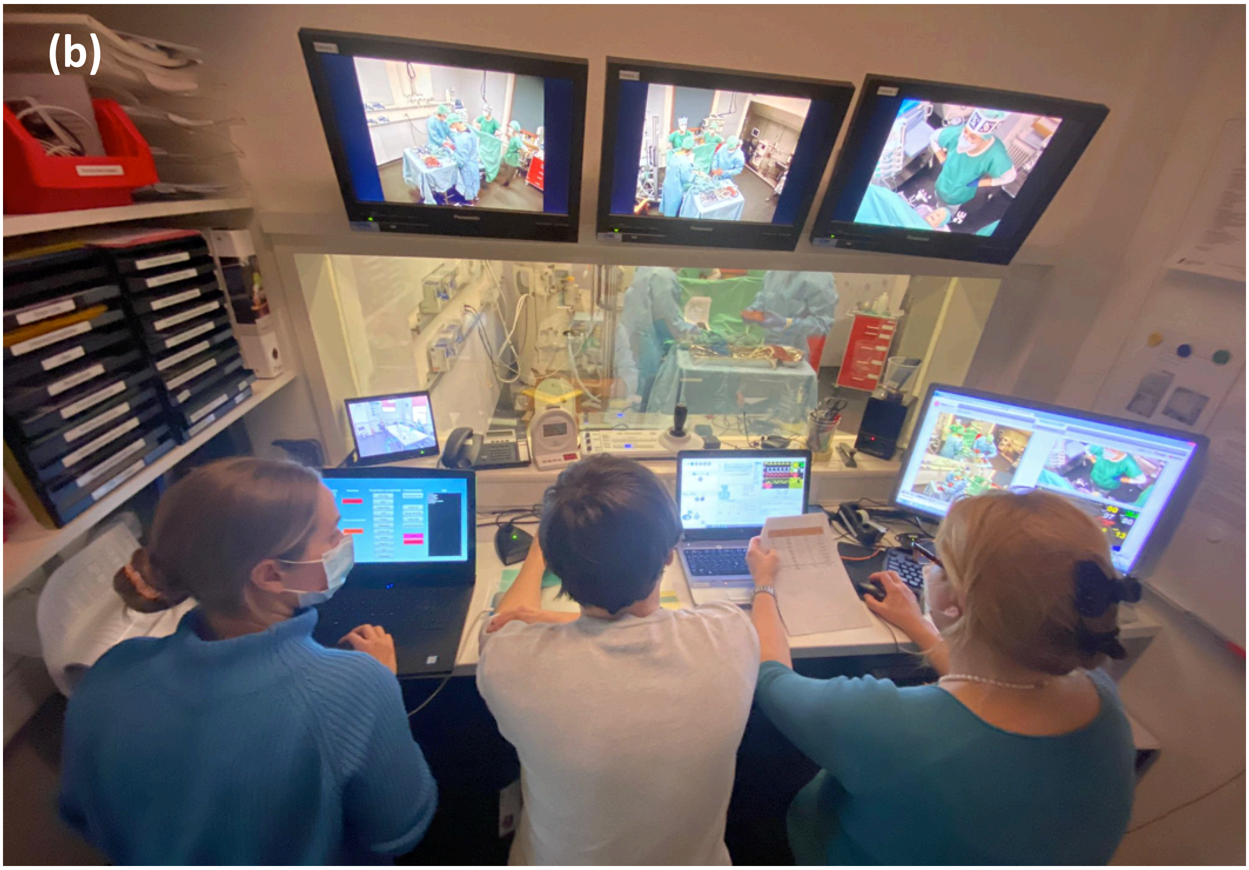

Supplement: Multimedia Appendix 4 [file jmir_v25i1e43895_app4.pdf]
