# Supplementary material for: Coagulation Management of Critically Bleeding Patients With Viscoelastic Testing Presented as a 3D-Animated Blood Clot (The Visual Clot): Randomized Controlled High-Fidelity Simulation Study
Source: J Med Internet Res. 2023 Oct 12;25:e43895. doi: 10.2196/43895 (PMC10603564; doi:10.2196/43895)
Supplement: Multimedia Appendix 5 [file jmir_v25i1e43895_app5.docx]

Multimedia Appendix 5

General Statements about Visual Clot Questionnaire

**Statement:**

With Visual Clot, I felt better prepared to interpret the result of a viscoelastic test.

**Possible Answers:**

Strongly agree; agree; disagree; strongly disagree.

**Statement:**

Visual Clot interpretation was easy to learn.

**Possible Answers:**

Strongly agree; agree; disagree; strongly disagree.

**Statement:**

I would use Visual Clot in daily clinical practice.

**Possible Answers:**

Strongly agree; agree; disagree; strongly disagree.

**Statement:**

Visual Clot seems too simplistic to be any good.

**Possible Answers:**

Strongly agree; agree; disagree; strongly disagree.
