# Supplementary material for: Coagulation Management of Critically Bleeding Patients With Viscoelastic Testing Presented as a 3D-Animated Blood Clot (The Visual Clot): Randomized Controlled High-Fidelity Simulation Study
Source: J Med Internet Res. 2023 Oct 12;25:e43895. doi: 10.2196/43895 (PMC10603564; doi:10.2196/43895)
Supplement: Multimedia Appendix 7 [file jmir_v25i1e43895_app7.pdf]

Simulation-Based Research Extensions for the CONSORT and STROBE Statement Checklist

From: Reporting guidelines for health care simulation research: extensions to the CONSORT and STROBE statements

Item	Item number	CONSORT/STROBE description (Observational Studies)	Extension for SBR	Completed: page number
Title and abstract	1	a. Indicate the study's design with a commonly used term in the title or the abstract.<br>b. Provide in the abstract an informative and balanced summary of what was done and what was found.	In abstract or key terms, the MESH or searchable keyword term must have the word simulation or simulated.	Page 1 and 2
Introduction				
Background/rationale	2	Explain the scientific background and rationale for the investigation being reported.	Clarify whether simulation is subject of research or investigational method for research.	Page 3
Objectives	3	State specific objectives, including any prespecified hypotheses.		Page 4
Methods				
Study design	4	Present key elements of study design early in the paper.		Page 5
Setting	5	Describe the setting, locations, and relevant dates, including periods of recruitment, exposure, follow-up, and data collection.		Page 5
Participants	6	a. Cohort study: give the eligibility criteria and the sources and methods of selection of participants. Describe methods of follow-up.<br>Case–control study: give the eligibility criteria and the sources and methods of case ascertainment and		Page 5

Item	Item number	CONSORT/STROBE description (Observational Studies)	Extension for SBR	Completed: page number
		control selection. Give the rationale for the choice of cases and controls.<br>Cross-sectional study: give the eligibility criteria and the sources and methods of selection of participants.<br>b. Cohort study: for matched studies, give matching criteria and number of exposed and unexposed.<br>Case–control study: for matched studies, give matching criteria and the number of controls per case.		
Variables	7	Clearly define all outcomes, exposures, predictors, potential confounders, and effect modifiers.<br>Give diagnostic criteria, if applicable.	Describe the theoretical and/or conceptual rationale for the design of the intervention/exposure.<br>Describe the intervention/exposure with sufficient detail to permit replication.<br>Clearly describe all simulation-specific exposures, potential confounders, and effect modifiers.	Page 6 and 7
Data sources/measurement	8	For each variable of interest, give sources of data and details of methods of assessment (measurement).<br>Describe comparability of assessment methods if there is >1 group.	In describing the details of methods of assessment, include (when applicable) the setting, instrument, simulator type, timing in relation to the intervention, along with any methods used to enhance the quality of measurements.<br>Provide evidence to support the validity and reliability of assessment tools in this context (if available).	Page 7 and 8
Bias	9	Describe any efforts to address potential sources of bias.		Page 10 to 12
Study size	10	Explain how the study size was arrived.		Page 12
Quantitative variables	11	Explain how quantitative variables were handled in the analyses. If applicable, describe which groupings were chosen and why.		Page 10 to 12

Item	Item number	CONSORT/STROBE description (Observational Studies)	Extension for SBR	Completed: page number
Statistical methods	12	a. Describe all statistical methods, including those used to control for confounding.<br>b. Describe any methods used to examine subgroups and interactions.<br>c. Explain how missing data were addressed.<br>d. Cohort study: if applicable, explain how loss to follow-up was addressed.<br>Case–control study: if applicable, explain how matching of cases and controls was addressed.<br>Cross-sectional study: if applicable, describe analytical methods taking account of sampling strategy.<br>e. Describe any sensitivity analyses.	Clearly indicate the unit of analysis (e.g., individual, team, system), identify repeated measures on subjects, and describe how these issues were addressed.	Page 10 to 12
Results				
Participants	13	a. Report the numbers of individuals at each stage of the study (e.g., numbers potentially eligible, examined for eligibility, confirmed eligible, included in the study, completing follow-up, and analyzed).<br>b. Give reasons for nonparticipation at each stage.<br>c. Consider use of a flow diagram.		Page 13
Descriptive data	14	a. Give characteristics of study participants (e.g., demographic, clinical, social) and information on exposures and potential confounders.<br>b. Indicate the number of participants with missing data for each variable of interest.<br>c. Cohort study: summarize follow-up time (e.g., average and total amount).	In describing characteristics of study participants, include their previous experience with simulation and other relevant features as related to the intervention(s).	Table 2
Outcome data	15	Cohort study: report numbers of outcome events or summary measures over time.		Page 13 to 15

Item	Item number	CONSORT/STROBE description (Observational Studies)	Extension for SBR	Completed: page number
		Case–control study: report numbers in each exposure category or summary measures of exposure.<br>Cross-sectional study: report numbers of outcome events or summary measures.		
Main results	16	a. Give unadjusted estimates and, if applicable, confounder-adjusted estimates and their precision (e.g., 95 % confidence intervals).<br>Make clear which confounders were adjusted for and why they were included.<br>b. Report category boundaries when continuous variables were categorized.<br>c. If relevant, consider translating estimates of relative risk into absolute risk for a meaningful time period.	For assessments involving >1 rater, interrater reliability should be reported.	Figure 3 and Page 13 to 15
Other analyses	17	Report other analyses done (e.g., analyses of subgroups and interactions and sensitivity analyses).		–
Discussion				
Key results	18	Summarize key results with reference to study objectives.		Page 16
Limitations	19	Discuss limitations of the study, taking into account sources of potential bias or imprecision. Discuss both direction and magnitude of any potential bias.	Specifically discuss the limitations of SBR.	Page 18 to 20
Interpretation	20	Give a cautious overall interpretation of results considering objectives, limitations, multiplicity of analyses, results from similar studies, and other relevant evidence.		Page 20

Item	Item number	CONSORT/STROBE description (Observational Studies)	Extension for SBR	Completed: page number
Generalizability	21	Discuss the generalizability (external validity) of the study results.	Describe generalizability of simulation-based outcomes to patient-based outcomes (if applicable).	Page 18
Other information				
Funding	22	Give the source of funding and the role of the funders for the present study and, if applicable, for the original study on which the present article is based.	List simulator brand and if conflict of interest for intellectual property exists.	Page 23
